# Repeated Positive Cervical HPV Testing and Absent or Minor Cytology Abnormality at Pap Smear. What is the Next Step?

**DOI:** 10.31557/APJCP.2021.22.6.1907

**Published:** 2021-06

**Authors:** Vitor Caeiro, Sara Nunes, Bruno Esteves, José Fonseca-Moutinho

**Affiliations:** 1 *Health Sciences Research Centre (CICS), Faculty of Health Sciences, University of Beira Interior (UBI), Covilha, Portugal. *; 2 *Polytechnic Institute of Castelo Branco, Castelo Branco, Portugal. *; 3 *Clinical Pathology at Cova da Beira University Hospital Center (CHUCB), Covilhã, Portugal.*; 4 *Health Sciences Research Centre (CICS), Faculty of Health Sciences, University of Beira Interior (UBI), Covilhã, Portugal. *

**Keywords:** Cervical cancer, HPV testing, cervical cancer screening, HSIL, CIN

## Abstract

**Background:**

Human papillomavirus (HPV) screening has significantly reduced cervical cancer (CC) mortality. Women who consecutively test positive for high-risk HPV without and minor changes on reflex cytology (atypical squamous cells of undetermined significance [ASC-US] or low-grade squamous intraepithelial lesion [LSIL]) or dysplasia on cervical colposcopy-oriented biopsy are always referred to colposcopy. The aim of the present study was to assess whether this guidance is appropriate for COBAS HPV testing with reflex cytology.

**Methods:**

A cross-sectional, retrospective study was carried out in 5,227 women who underwent routine CC screening over a period of five years (2012-2017). All HPV tests were performed using Cobas®4800 HPV. The study included women attending gynecology appointments whose first HPV test was positive and who had any type of follow-up. Patients’ HPV test results as well as cytology and biopsy findings obtained during the abovementioned period were analyzed. A descriptive and comparative statistical study was conducted using this data.

**Results:**

A total of 765 out of 6003 HPV tests performed in 5,227 women were positive, and 141 women who had a positive HPV test (with negative for intraepithelial lesion or malignancy [NILM] or inflammation, or ASC-US and LSIL cytology, but no lesions on colposcopy, or absence of dysplasia on histology) repeated the HPV test at least once. Of these 141 women, 6 were diagnosed with high-grade squamous intraepithelial lesion (HSIL) during the follow-up period. All cases of HSIL were diagnosed after the second HPV test.

**Conclusion:**

This study shows that, at cervical cancer screening, all women testing positive for HPV regardless of Pap smear result should be referred to colposcopy.

## Introduction

According to the World Health Organization (WHO) statistics, around 15 to 20% of the diagnosed cancers are associated with viral infections. Human papillomavirus (HPV) is one of the viruses contributing to these statistics, increasing the risk of cervical cancer (CC) progression when high-risk HPV infection persists (Chan et al., 2019). CC is the fourth most common cancer in women worldwide, after breast cancer, colorectal cancer and lung cancer (Bhatla and Denny, 2018).

Screening programs which incorporate HPV testing have consistently been associated with a reduction in CC incidence, potentially decreasing morbidity and mortality (Chan et al., 2019). Nevertheless, CC remains a major public health problem, with estimated 569,847 new cases and 311,365 deaths worldwide in 2018 (Bray et al., 2018).

Persistent infection with high-risk HPV genotypes is a necessary but not sufficient condition for disease progression and is the main epidemiological driver of high-grade intraepithelial lesions (HSIL) and invasive carcinoma (Oliveira et al., 2013). HPV infection is subclinical in most cases, especially in younger women where in more than 80% of cases the infection resolves spontaneously within 1 to 2 years. However, approximately 10% of HPV infections can become persistent and about 3 to 4% progress to intraepithelial lesions. Of these, 0.7 to 1% may advance to high-grade lesions (CIN 2/3), being estimated that 0.1% will progress to invasive cancer if not detected and treated in a timely manner (WHO, 2012).

The natural course of CC is well known, and its carcinogenesis process is slow. The presence of CC precursor lesions, the availability of sensitive screening tests for detection and effective treatment methods have enabled highly effective secondary prevention, using screening programs (Tsikouras et al., 2016).

The most common CC screening methods are conventional cytology, liquid-based cytology and HPV testing, or an association of the latter two (WHO, 2012). The “standard” screening method has been morphological cytology. Several studies have shown that HPV testing is more sensitive than cytology alone in detecting and preventing high-grade lesions and progression to cancer. In addition, when using HPV testing as a screening method, the presence of a negative test allows the screening interval to be extended to 5 years, improving compliance with screening programs and enabling effective cost reductions of approximately 20% (Schiffman et al., 2011; Agorastos et al., 2015; Goodman, 2015; Tsikouras et al., 2016).

In 2017, a national organized CC HPV-based screening program was implemented in Portugal for women between the ages of 25 and 60 years, performed every 5 years, with reflex cytology for high-risk HPV genotypes other than HPV 16 and 18. This screening program introduces updates to the previous regional cervical cancer screening programs and states that women with a positive HPV test for genotypes other than 16 and 18 with negative for intraepithelial lesion or malignancy (NILM) cytology should repeat the HPV test within the following year. In case of a second HPV test is positive, the woman will be referred for colposcopy. Following 2013 Kaiser Permanent Northern California (KPNC) study results, women with a repetitive positive HPV test with without or minor cytological abnormality (ASC-US/LSIL) and no dysplasia on cervical oriented-colposcopy biopsy should be recommended to colposcopy based on co-testing and Hybrid Capture 2 (HC2; Qliagen, Germantown, MD) for HPV testing (Katki et al., 2013). However, no scientific report showed whether this approach is useful on cervical cancer screenings based on primary new molecular technologies for HPV testing with reflex cytology.

Our goal was to use the opportunistic CC screening program of the Cova da Beira University Hospital Center (CHUCB), based on primary COBAS HPV testing and triage cytology, to validate colposcopy recommendation for those women with repeated positive HPV test for genotypes other than HPV 16 and 18 and NILM or minor lesions on previous cytology and no previous dysplasia detected on cervical oriented-colposcopy biopsy.

## Materials and Methods

A cross-sectional and retrospective study was carried out based on data from the routine CC screening protocol in force at CHUCB between August 2012 and August 2017. The screening protocol was based on HPV testing as the primary method for all women over 25 years old with no history of CC screening in the past 2 years who attend gynecology appointments at the CHUCB.

The screening method was the Cobas®4800 HPV test, which detects HPV 16, HPV 18 and other types of HPV (31, 33, 35, 39, 45, 51, 52, 56, 58, 59, 66, 68), using the liquid medium Surepath^®^.

All HPV tests were performed at the Laboratory of the Clinical Pathology Department of the CHUCB, while the cytological and histological tests were conducted at the Anatomical Pathology Department of the CHUCB.

**Figure 1.**
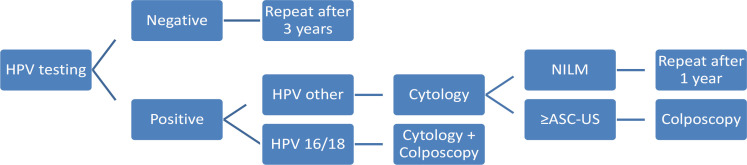
Flowchart of Cervical Cancer Screening Implemented on Cova da Beira University Hospital Center (CHUCB). A negative HPV test should be repeated after 3 years. However, women with HPV positive test for other strains than 16 or 18 are examined by reflex cytology. In case of negative for intraepithelial lesion or malignancy (NLIM), cytology should be repeated after one year. Otherwise, women should be referred to colposcopy. In case of infection by HPV 16 or 18 genotypes, the follow-up incorporates both cytology and colposcopy

The CC screening program of the CHUCB was designed and implemented by the CHUCB Colposcopy Unit, where all colposcopic examination were performed. According to the CHUCB screening algorithm, shown in Figure 1, a negative HPV tests should be repeated after 3 years; a positive HPV test for genotypes 16 or 18 is followed by reflex cytology and referral for a colposcopy; a positive HPV test for other types of HPV is followed by reflex cytology, and if reflex cytology shows NILM, the test must be repeated after 1 year; any other cytological finding requires referral for a colposcopy. After a second consecutive positive HPV test, the woman is monitored at the Colposcopy Unit for at least 3 years, regardless of subsequent HPV test results and cytology findings.

Of all 6,003 HPV tests performed in 5,227 women who underwent routine screening at the CHUCB over the abovementioned 5 years, 765 (14.6%) women who had a positive HPV test were selected for our study. Of these, we evaluated 141 women who had no history of treatment for cervical intraepithelial neoplasia and had satisfactory cytology findings, classified as NILM or minor cytological lesions (ASC-US/LSIL), normal colposcopy and/or no dysplasia on biopsy, and who had follow-up appointments at the Colposcopy Unit of the CHUCB. A biopsy was required during a colposcopy appointment only in the presence of grade 1 or 2 colposcopic findings or signs of invasion. If the transformation zone is classified as type 3 (squamouscolumnar junction not fully visible), endocervical curettage is performed routinely.

A descriptive statistical analysis of the data was performed, using the IBM SPSS application software, version 26 (SPSS Inc., Chicago, IL). In all cases, we analyzed the patient’s age, HPV test results (type 16, 18 and others), and cytology and histology findings of the biopsy obtained using colposcopy.

**Table 1:** Sequency of Results Human Papillomavirus (HPV) Tests, Cytology and Histology Results. Data is presented as number (percentage, %).

	1^st^ Test (n=141)	2^nd^ Test (n=141)	3^rd^ Test (n=55)	4^th^ Test (n=19)	5^th^ Test (n=6)
HPV test					
Negative	-	61 (43.3)	20 (36.4)	4 (21.1)	3 (50)
HPV 16	16 (11.3)	6 (4.2)	2 (3.6)	1 (5.3)	1 (16.6)
HPV 18	5 (3.5)	-	1 (1.8)	-	-
Others	102 (72)	59 (41.8)	26 (47.3)	11 (57.9)	2 (33.4)
HPV 16+others	15 (10.6)	11 (7.8)	5 (9.1)	2 (10.6)	-
HPV 18+others	3 (2.1)	3 (2.1)	1 (1.8)	1 (5.3)	-
CYTOLOGY					
Not performed	1 (0.7)	55(39)	17(31)	1(5.3)	3(50)
NILM	101 (71.7)	51 (36.2)	28 (50.9)	13 (68.4)	2 (33.3)
LSIL	24 (17.0)	13 (9.3)	3 (5.4)	2 (10.5)	-
ASC-US	15 (10.6)	15 (10.6)	6 (10.9)	2 (10.5)	1 (16.7)
HSIL	-	3 (2.1)	1 (1.8)	1 (5.3)	-
ASC-H	-	4 (2.8)	-	-	-
HISTOLOGY					
Not performed	24 (53.3)	104 (73.7)	49 (89.1)	14 (73.7)	4 (66.6)
No dysplasia	21 (46.7)	18 (12.8)	4 (7.3)	3 (15.8)	1 (16.7)
LSIL	-	13 (9.2)	2 (3.6)	2 (10.5)	1 (16.7)
HSIL	-	6 (4.3)	-	-	-

ASC-H, atypical squamous cells; ASC-US, atypical squamous cells of undetermined significance; HPV, human papillomavirus; HSIL, high-grade squamous intraepithelial lesion; LSIL, low-grade squamous intraepithelial lesion; NILM, negative for intraepithelial lesion or malignancy.

**Table 2:** Sequency of Human Papillomavirus (HPV) Tests, Cytology and Histology Results Comparing Women under and over 30 Years of Age. Data is presented as number (percentage, %).

	1^st^ Test		2^nd^ Test		3^rd^ Test		4^th^ Test		5^th^ Test	
Age	<30	>30	<30	>30	<30	>30	<30	>30	<30	>30
Number of cases	30 (21.3)	111 (78.7)	30 (21.3)	111 (78.7)	11 (20)	44 (80)	2 (1.1)	17 (98.9)	-	6 (100)
HPV TEST										
Negative	-	-	15 (50)	46 (41.4)	4 (36.4)	16 (36.3)	1 (50)	3 (17.6)	-	3 (50)
HPV 16	8 (26.7)	8 (7.2)	1 (3.3)	5 (4.5)	1 (9.1)	1 (2.3)	-	1 (5.9)	-	1 (16.7)
HPV 18	-	5 (4.5)	-	-	1 (9.1)	-	-	-	-	-
Others	19 (63.3)	83 (74.8)	9 (30)	50 (45)	5 (45.4)	21 (47.7)	1 (50)	10 (58.8)	-	2 (33.3)
HPV 16+HPV 18	-	-	-	1 (0.9)	-	-	-	-	-	-
HPV 16+others	3 (10)	12 (10.1)	4 (13.3)	7 (6.3)	-	5 (11.4)	-	2 (11.8)	-	-
HPV 18+others	-	3 (2.7)	1 (3.3)	2 (1.8)	-	1 (2.3)	-	1 (5.9)	-	-
CYTOLOGY										
Not performed	1 (3.3)	-	14 (46.7)	41 (37)	3 (27.3)	14 (31.8)	1 (50)	-	-	3 (50)
NILM	20 (66.7)	81 (73)	12 (40)	39 (35	8 (72.7)	20 (45.5)	1 (50)	12 (70.6)	-	2 (33.3)
LSIL	6 (20)	18 (16.2)	2 (6.7)	11 (10)	-	3 (6.8)	-	2 (11.8)	-	-
ASC-US	3 (10)	12 (10.8)	1 (3.3)	14 (12.6)	-	6 (13.6)	-	2 (11.8)	-	1 (16.7)
HSIL	-	-	1 (3.3)	2 (1.8)	-	1 (2.3)	-	1 (5.8)	-	-
ASC-H	-	-	-	4 (3.6)	-	-	-	-	-	-
HISTOLOGY										
Not performed	6 (20)	18 (16.2)	24 (80)	80 (72)	9 (81.8)	40 (91)	1 (50)	12 (70.6)	-	4 (66.6)
No dysplasia	3 (10)	18 (16.2)	2 (6.7)	14 (12.6)	2 (18.2)	2 (4.5)	1 (50)	3 (17.6)	-	1 (16.7)
LSIL	-	-	-	13 (11.7)	-	2 (4.5)	-	2 (11.8)	-	1 (16.7)
HSIL	-	-	2 (6.7)	4 (3.6)	-	-	-	-	-	-

ASC-H, atypical squamous cells; ASC-US, atypical squamous cells of undetermined significance; HPV, human papillomavirus; HSIL, high-grade squamous intraepithelial lesion; LSIL, low-grade squamous intraepithelial lesion; NILM, negative for intraepithelial lesion or malignancy.

**Table 3:** Detailed Description of High-Grade Squamous Intraepithelial Lesion (Cases of HSIL) Diagnosed during Study Follow-up

Description of positive cases						
Age	1^st^ HPV Test	1^st^ Cytology	2^nd^ HPV Test	2^nd^ Cytology	Time to diagnosis	Notes
25 years old	16 + Others	NILM	Others	HSIL	18 months	
27 years old	16 + Others	LSIL	16 + Others	LSIL	22 months	1)
30 years old	Others	NILM	Others	HSIL	25 months	
40 years old	Others	NILM	Others	NILM	16 months	2)
42 years old	Others	LSIL	Others	ASC-US	14 months	
50 years old	Others	LSIL	Negative	ASC-US	16 months	3)

ASC-US, atypical squamous cells of undetermined significance; HPV, human papillomavirus; HSIL, high-grade squamous intraepithelial lesion; LSIL, low-grade squamous intraepithelial lesion; NILM, negative for intraepithelial lesion or malignancy; 1) Despite LSIL cytology, the patient underwent colposcopy and biopsy revealing HSIL; 2) Despite NILM cytology, the patient underwent colposcopy and biopsy revealing HSIL; 3) Co-testing (HPV testing + cytology) Patient underwent colposcopy and biopsy revealing HSIL

## Results

The study sample consisted of 141 women who had a positive HPV test (to HPV 16, 18 and others) with reflex cytology classified as NILM or minor cytological lesions (ASC-US/LSIL), but normal colposcopy and/or no dysplasia on biopsy and who underwent follow-up (Table 1). This corresponds to 18.4% of all women with a positive HPV test during the study, aged between 17 and 69, with a mean age of 39.3 years (standard deviation=11.1). For these women, the mean follow-up was 36.6 months (standard deviation=18.5). 

During follow-up, CIN2+ lesions were detected in six (4.3%) women, with a mean age of 35.7 years (standard deviation= 7.7), and all CIN2+ lesions were diagnosed after the second HPV test. No women were diagnosed with invasive carcinoma. The mean time to diagnosis of CIN2+ lesions was 18.5 months (standard deviation=4.2). The HPV test, cervical cytology and biopsy results are shown in Table 1. Regression rate of HPV infection in the studied group was always very high, especially for types 16 and 18, which highlights the transient nature of those HPV infections. However, the multiple infection rate (HPV 16 or 18 and others) remained unchanged, possibly due to reinfection. Following the first test, only 45 women underwent colposcopy due to a positive HPV 16 or 18 test and/or ASC-US or LSIL cytology. All women underwent colposcopy in their second, third, fourth and fifth HPV tests. Six women were co-tested for their second HPV test, and 3 women were co-tested for their third and fourth tests.

Table 2 shows that the prevalence and spontaneous resolution of high-risk HPV infection was more common in women under 30 years of age, while the cytological and histological diagnosis was more serious in the group of women over 30 years of age.

Table 3 shows the relevant aspects of the 6 cases where HSIL was diagnosed during patient follow-up. Four of these 6 cases were diagnosed in women aged 30 or over. HSIL was associated with HPV 16 infection in only one woman, and cytology had been classified as NILM or ASC-US or LSIL in 4 women.

## Discussion

The protocol used in this study was the CC screening protocol of the Gynecology Department of the CHUCB, which recommends HPV testing as the primary test in routine screening. This is an institutional screening program which, among other aspects, is open to all patients attending gynecology appointments (including pregnant women) and had the participation of all physicians who offer gynecology appointments at the CHUCB. This cervical cancer screening was implemented at the CHUCB to manage patients and to mitigate the effects of low compliance with the national screening program.

The CC screening protocol at the Gynecology Department of the CHUCB beginning at 2012 was organized following 2011 ATHENA HPV study results (Wright et al., 2012).

A high percentage of women under the age of 30 were included in the study population. The CC screening protocol in force at the CHUCB includes women over 25 years of age and some physicians did not comply with the inclusion criteria. There was a higher prevalence and spontaneous resolution of high-risk HPV infection in the group of women under 30 years of age, as well as less serious cytological and histological diagnoses, which is in accordance with the literature.

The HPV test used for screening was the Cobas^®^4800 HPV test, which is a qualitative test that uses real-time PCR technology to simultaneously detect DNA from 12 types of human recombinant HPV (31, 33, 35, 39, 45, 51, 52, 56, 58, 59, 66, and 68) and individually detect HPV 16 and 18. β-globin gene is amplified as an internal control. It can be used as a primary screening method with reflex cytology for positive HPV or in addition to cytology (co-testing). Thus, CIN3+ risk stratification is improved, increasing sensitivity for early detection of cervical cancer, with a negative predictive value very close to 100% (Chan et al., 2019).

The liquid medium used for transport and preservation of all samples for cytology was SurePath^®^, which does not exhibit significant differences in terms of cut-off values, when compared to other certified liquid collection media, for the detection of CIN1, CIN2+ and CC lesions (Rozemeijer et al., 2016).

Some women who had a positive HPV test did not undergo follow-up because they had a surgery for a benign condition (uterine fibroids or pelvic organ prolapse corrections), or they stopped attending appointments. 

In accordance with literature, the percentage of multiple infections was different according to age, suggesting transient reinfection rather than a persistent infection (Pista et al., 2011).

From the analysis of the 6 cases of HSIL diagnosed, we highlight the importance of performing colposcopy after the second test, as all our cases were diagnosed at this time. Our results are validated by other studies reporting similar situations (Melnikow et al., 2018; Gu et al., 2019). The second cytology was suggestive of HSIL in only two cases, and it was classified as NILM in one case, which reinforces the value of colposcopy in these situations.

The absence of HSIL diagnosed after the second HPV test is probably due to the referral for colposcopy of all patients after the second positive HPV test, regardless of cytology findings. This procedure allowed HSIL identification which was not diagnosed during the first test. Between the first and second tests, it is more likely that there was regression than progression of dysplastic lesions, which may also explain in part why no other cases of HSIL were diagnosed after the second test. The outcome of any HPV-based CC screening is highly dependent on the number of lesions detected using colposcopy-directed biopsy, which reinforces the importance of quality colposcopy practices. Avoiding unnecessary biopsies without neglecting the diagnosis of cervical cancer precursor lesions is of paramount importance, and all women with positive HPV tests should be referred to different colposcopy units, as was the case in this study.

This study demonstrates that women undergoing HPV-based CC screening who had one positive HPV test with NILM, ASC-US or LSIL cytology, with normal colposcopic findings and/or no dysplasia on cervical biopsy, should be referred for colposcopy in the presence of a second positive HPV test, regardless of the cytology findings. This procedure is standardized in the current cervical cancer screening program in Portugal and recommended by 2019 American Society for Colposcopy and Cervical Pathology (ASCCP) guidelines (Perkins et al., 2020).

This study has some limitations. The CC screening method used at the CHUCB is an institutional routine program based on a random population that attends gynecology appointments and includes pregnant women, and it is not an organized screening program. Furthermore, sample size is limited as the geographic localization of CHUCB only serves a population of approximately 90,000 people which includes the municipalities of Covilhã, Fundão, Belmonte and Penamacor. Only women referred by CHUCB physicians to the CHUCB Colposcopy Unit were evaluated in this study. Many of these women had previously participated in the organized cervical cancer-screening program in the Centre Region of Portugal, which has been in place for more than 20 years. The influence of HPV vaccination on the results was not evaluated because the percentage of vaccinated women was small at the time of data collection.

Nevertheless, the results of our study concerning HPV and cytology abnormalities prevalence are in agreement with studies performed in other countries, such as the HEllenic Real life Multicentric cErvical Screening study group, in Greece (HERMES) (Agorastos et al., 2015) and a study that evaluates the efficacy outcomes of primary HPV testing based on the follow-up of randomized controlled trials in Germany (WOLPHSCREEN), Sweden (SWEDESCREEN), England (ARTISTIC), the Netherlands (POBASCAM) and Italy (NTCC) (Ronco et al., 2014). Therefore, we can conclude that our studied population was adequate for valid conclusions. In addition, our study is in agreement with recent published 2019 ASCCP Risk-Based Management Consensus Guidelines for Abnormal Cervical Cancer Screening Tests and Cancer Precursors (Perkins et al., 2020), that recommend colposcopies for all women with repeated positive HPV testing with NILM or minor cytology abnormalities.

This study has shown that, regardless of reflex cytology findings, women who have at least two consecutive positive cervical HPV tests are at increased risk of having previously undiagnosed cervical HSIL and should always be referred for colposcopy. Additionally, the risk of intraepithelial lesions or malignancy was independent of the type of HPV determined. All women with cervical repeated positive HPV testing and with absent or minor cytology abnormalities should be referred to colposcopy in an independent way of screening adopted program and technology used for HPV testing, as recommended by ASCCP.

*Abbreviations*

ASCCP – American Society for Colposcopy and Cervical Pathology; ASC-H – atypical squamous cells; ASC-US – atypical squamous cells of undetermined significance; CC – cervical cancer; CHUCB – Cova da Beira University Hospital Center; CIN – cervical intraepithelial neoplasia; Co-testing: Concomitant HPV test and cytology; HPV – human papillomavirus; HSIL – high-grade squamous intraepithelial lesion; KPNC – Kaiser Permanent Northern California; LSIL – low-grade squamous intraepithelial lesion; NILM – negative for intraepithelial lesion or malignancy; SPSS – Statistical Package for the Social Sciences.

## Author Contribution Statement

Vitor Caeiro: Conducted the investigation and write manuscript; Sara Nunes: Organize statistical analysis of data and review the manuscript; Bruno Esteves: Responsible by HPV testing and pap smear and review the manuscript; José Fonseca-Moutinho; Advisor and review the manuscript.
